# Catheter Ablation of atrial fibrillation vs. atrioventricular nodal ablation with Conduction system pacing in persistent atrial fibrillation and heart failure (ABACUS): rationale and design

**DOI:** 10.1093/ehjopen/oeag007

**Published:** 2026-01-19

**Authors:** Haran Burri, Nikola Kozhuharov, Kerstin Bode, Oscar Cano, Karol Čurila, Inga Drossart, Laszlo Geller, Hein Heidbuchel, Marek Jastrzebski, Jarkko Karvonen, Christophe Leclercq, José L Merino, Helmut Pürerfellner, Jan Tijssen, Vassil Traykov, Isabelle van Gelder, Kevin Vernooy, Zachary Whinnett, Michele Brignole

**Affiliations:** Cardiology Department, University Hospital of Geneva 4, Rue Gabrielle Perret Gentil, 1211 Geneva, Switzerland; Cardiology Department, Inselspital Bern, Freiburgstrasse 20, 3010 Bern, Switzerland; Heart Center Leipzig at University of Leipzig, Department of Electrophysiology, Strümpellstraße 39, 04289 Leipzig, Germany; Hospital Universitario y Politecnico La Fe, Avinguda de Fernando Abril Martorell, 106, Quatre Carreres, 46026 Valencia, Spain; University Hospital, Kralovske Vinohrady, Šrobárova 1150 /50, 100 00 Prague, Czech Republic; European Society of Cardiology Patient Forum, Sophia Antipolis,2035 Rte des Colles, 06410 Biot, France; Semmelweis University, Üllői út 26, 1085 Budapest, Hungary; Antwerp University Hospital, Drie Eikenstraat 655, 2650 Edegem, Antwerp, Belgium; Cardiovascular Research, GENCOR, Antwerp University, 132000 Prinsstraat, GENCOR, Antwerp University, 2000 Antwerp, Belgium; Jagiellonian University, Jakubowskiego 2, Krakow 30-688, Poland; Heart and Lung Center, Helsinki University Hospital and University of Helsinki, Tukholmankatu 8 U, 00290 Helsinki, Finland; Cardiology Department, CHU Rennes, 2 Rue Henri le Guilloux, 35000 Rennes, France; Hospital Universitario La Paz, IdiPaz, 261 P.º de la Castellana, 28046 Madrid, Spain; Ordensklinikum Linz, Elisabethinen, Fadingerstraße 1, 4020 Linz, Austria; AMC Amsterdam, Meibergdreef 9, 1105 AZ Amsterdam, the Netherlands; Department of Invasive Electrophysiology, Acibadem City Clinic, Tokuda University Hospital, 51 Nikola Vaptsarov Blvd., 1407 Sofia, Bulgaria; Cardiology Department, University Medical Center Groningen, Hanzeplein 1, 9713 GZ Groningen, the Netherlands; Department of Cardiology, Cardiovascular Research Institute Maastricht (CARIM), Maastricht University Medical Center, Universiteitssingel 40, 6229 ER Maastricht, the Netherlands; Cardiology Department, National Heart and Lung Institute, Imperial College London, South Kensington, London SW7 2AZ, UK; Department of Cardiology, IRCCS Istituto Auxologico Italiano, Faint and Fall Research Centre, San Luca Hospital, Milan, Italy

**Keywords:** Persistent atrial fibrillation, Heart failure, Catheter ablation, Conduction system pacing, Atrioventricular nodal ablation, Pace and ablate

## Abstract

**Aims:**

Patients with persistent atrial fibrillation (AF) and heart failure (HF) have compromised clinical outcomes. Contemporary management includes rhythm control with AF ablation, or rate control and regularization with conduction system pacing and atrioventricular nodal ablation (CSP + AVNA). These strategies have never been compared in a randomized clinical trial. The study aims to determine whether CSP + AVNA is superior to AF ablation for reducing all-cause mortality and cardiovascular hospitalization, and noninferior with respect to all-cause mortality and heart failure hospitalization.

**Methods and results:**

ABACUS is a multicentre, investigator-initiated, randomized controlled trial enrolling 220 patients with persistent AF and HF, aged >60 years, who are eligible for both treatment modalities, with at most one previous AF ablation procedure. Participants will be randomized 1:1 to either catheter ablation of AF (with pulmonary vein isolation using any routine technique) or to CSP + AVNA. All patients will undergo at least one year of follow-up. The co-primary endpoints will be tested sequentially. A number of predefined secondary endpoints, including costs, will also be evaluated.

**Discussion:**

ABACUS compares CSP + AVNA with AF ablation in patients with persistent AF and HF. The results will provide evidence to improve care in this vulnerable patient population.

## Introduction

Atrial fibrillation (AF) impacts heart function by loss of atrial contractile function, induction of tachycardiomyopathy, and deterioration of cardiac pump function due to irregular and often short cycles.^1–5^ The coexistence of persistent AF with heart failure (HF) confers an adverse prognosis.^6^

A number of strategies have been studied for treating HF patients with AF, and are summarized in *Table 1*. Maintenance of sinus rhythm by antiarrhythmic drugs has not been shown to be superior to pharmacological rate control.^9,11,30^ Catheter ablation of AF with pulmonary vein isolation (PVI) is well established^31^ and has been shown to be superior in terms of clinical endpoints as compared to medical rate or rhythm control,^12,14,16,20,22^ although some trials were negative.^15,22^ However, all these trials were performed before the era of pulsed field ablation (PFA), which has been shown to be safe and effective, although data in the HF population with persistent AF are still sparse, with 1-year recurrence rates of atrial arrhythmias of about 35%.^32^

**Table 1:** Randomized trials for treating atrial fibrillation in patients with heart failure comparing different treatment strategies

Comparison	Drug rate control	Drug rhythm control	AVNA + RVP	AVNA + BiV
Drug rhythm control	AFFIRM^7^		AIRCRAFT^8^	-
	AF-CHF^9^		Brignole 1998^10^	
	RACE^11^			
AF ablation	CAMTAF^12^		-	PABA-CHF^13^
	CASTLE-AF^14^			
	AMICA^15^	AATAC^16^		
	CAMERA-MRI^17^	AMICA^15^		
	ARC-HF^18^	CASTLE-AF^14^		
	MacDonald et al^19^	CASTLE-HTx^20^		
	CABANA^21^			
	RAFT-AF^22^			
AVNA + BiV	APAF-CRT^23,24^	-	APAF^25^	
			PAVE^26^	
			Brignole 2005^27^	
			AVAIL CLS/CRT^28^	
AVNA + HBP	-	-	-	ALTERNATIVE-AF^29^

Abbreviations: AF, atrial fibrillation; AVNA, atrioventricular nodal ablation; BiV, biventricular pacing; HBP, His bundle pacing; RVP, right ventricular pacing.

Another approach is ‘pace and ablate’ for which atrioventricular nodal ablation (AVNA) combined with right ventricular pacing (RVP) or biventricular pacing (BiVP) has been found to be superior to pharmacological therapy in patients with permanent AF with HF or AF symptoms.^8,10,23,24^ For this strategy, BiVP has been shown to provide better outcomes than RVP.^25,26^ Ablate and pace has been tested across the spectrum of left ventricular systolic function with an average left ventricular ejection fraction (LVEF) of approximately 40% in the APAF and APAF-CRT trials.^23,25^ More recently, conduction system pacing (CSP) with either His bundle pacing (HBP) or left bundle branch area pacing (LBBAP) have emerged as being more physiological alternatives to these standard pacing modalities.^33–36^ A small crossover study on AVNA randomized HBP against BiVP in patients with a narrow QRS or right bundle branch block and found superior left ventricular ejection fraction (LVEF) with HBP.^29^

The only study that randomized PVI against a ‘pace and ablate’ strategy utilized BiVP and showed that PVI was superior in terms of combined soft endpoints (LVEF, distance on the 6-minute walk test, and quality of life).^13^ However, only about half of the population had persistent AF, with an average heart rate of 80bpm and QRS width of 90 ms (and it is known that BiVP can be detrimental in patients with a narrow QRS and heart failure.^37^)

There are currently no trials which have randomized PVI against a ‘pace and ablate’ strategy with CSP in patients with persistent AF and HF. Both strategies have their advantages and limitations. Restoring and maintaining sinus rhythm by catheter ablation can improve HF due to regularization of cardiac rhythm and the restoration of atrial contractile function. However, atrial contractile dysfunction may persist in scarred atria (in which ablation results in further fibrosis). Furthermore, recurrence rates with radiofrequency ablation are high at approximately 50% at 12–18 months^38,39^ and the procedure is associated with risks as well as with significant costs. A ‘pace and ablate’ strategy is a simpler alternative which can improve HF by reversing tachycardiomyopathy, regularizing cardiac rhythm, and eliminating the need for rate-slowing drugs which may have a detrimental effect on cardiac function. However, it usually condemns the patient to AF with loss of atrial contractile function and physiological rate response (although it has been reported that 13% of patients with AF since >6 months revert spontaneously to sinus rhythm after AVNA with CSP^40^) and results in pacemaker dependency (although most patients retain an escape rhythm.^41^)

The increasing prevalence of persistent AF and HF is a challenge to the healthcare system to meet the demand for treatment, both in terms of work burden as well as cost. As previously mentioned, both PVI and ‘pace and ablate’ have been shown to be superior to pharmacological treatment, but it is ill-defined whether one of these two modalities is superior. The clinical conundrum is also present in patients who have undergone one previous attempt at PVI and in whom AF has recurred, in whom a repeat ablation needs to be weighed against a ‘pace and ablate’ option. Therefore, there is a need to compare these interventions head-to-head in their contemporary form to better define treatment strategies in this patient population.

## Methods

### Objectives

The study has two primary goals: to show that the CSP + AVNA strategy is (i) superior to AF ablation in terms of reducing all-cause mortality and cardiovascular hospitalization (CVH), and (ii) noninferior with respect to all-cause mortality and heart failure hospitalization (HFH).

### Study description

The ABACUS trial is an investigator-initiated, international, multicentric, randomized, controlled, and open-label, two-arm comparative clinical trial. It is a head-to-head comparison of AF ablation (the control arm) to CSP + AVNA in patients with persistent AF and HF who are considered eligible for both treatments. Due to the nature of the interventions, it is not possible to blind patients and primary investigators.

The trial was designed by a steering committee composed of experts in the fields of AF, cardiac pacing, catheter ablation, and HF, as well as a biostatistician (J.T) and a patient representative (I.D). The trial is conducted in academic hospitals in the following 14 countries across Europe in 30 centres (with a maximum of four centres per country to provide geographical balance): Austria, Belgium, Bulgaria, the Czech Republic, Finland, France, Germany, Hungary, Italy, Poland, Spain, Switzerland, the Netherlands, and the UK. The sponsor is the University Hospital of Geneva, Switzerland, and the trial is funded by the Swiss National Science Foundation (https://data.snf.ch/grants/grant/220116) and the GeCOR Research Foundation of the Department of Cardiology at the University Hospital (https://cardiologie-universitaire-geneve.ch/).

Logistical support and monitoring are conducted by the Clinical Research Organization (PRODUCT—project development consulting, Sofia, Bulgaria). The database is managed by the Clinical Trial Unit of the University Hospital Geneva.

The study has been registered on www.clinicaltrials.gov under the number NCT06207383, in the ISRCTN registry under number ISRCTN65526476, and on the Swiss National Clinical Trial Portal under number SNCTP000006064.

This study is conducted in compliance with the Declaration of Helsinki, the ICH-GCP as well as other locally relevant legal and regulatory requirements.

#### Patient population

Consecutive subjects who are eligible for the standard treatment of catheter ablation of persistent AF will be considered for inclusion in the trial. Inclusion and exclusion criteria are listed in *Table 2*. Patients who are considered poor candidates in routine clinical practise for one of the treatment modalities (e.g. persistant AF for several years with a severely dilated left atrium who are unlikely to benefit from AF ablation) or who are unsuitable for one of the therapies (e.g. tricuspid valve prosthesis, which contraindicates CSP) will not be included.

**Table 2:** Inclusion and exclusion criteria for the ABACUS trial

**Inclusion criteria**
Subjects fulfilling all of the following criteria are eligible for the investigation:
Age ≥ 60 years. Persistent AF with symptomatic HF despite medical therapy, considered to be suitable for AF ablation, *with at most one previous PVI procedure*. Persistent AF is defined as AF that is continuously sustained beyond seven days, including episodes terminated by cardioversion (drugs or electrical) after > 7 days.^42^ At least one prior hospital admission, or emergency room/HF clinic visit for HF in the past 2 years, with BNP > 250 pg/mL or NT-pro-BNP > 1000 pg/mL measured at any timepoint during this interval. Previous or current rate or rhythm control drug therapy. Considered eligible for CSP implantation as an alternative to AF ablation Informed Consent signed by the subject.
**Exclusion criteria**
The presence of any one of the following criteria will lead to the exclusion of the subject:
NYHA Class IV and systolic blood pressure ≤80 mmHg despite optimized therapy. Life expectancy < 2 years. Need for major surgical intervention. Myocardial infarction, stroke, or PCI within the previous 3 months. Previously implanted or planned implantation of a CRT device or pacemaker. ICD implantation without a pacing indication is acceptable. Participation in another controlled trial with active treatment. Inability to sign an informed consent form

Abbreviations: AF, atrial fibrillation; BNP, brain natriuretic peptide; CRT, cardiac resynchronization therapy; ICD, Implantable cardioverter defibrillator; HF, heart failure; NYHA, New York Heart Association; PVI, pulmonary vein isolation.

If a patient has a standard indication for an implantable cardioverter defibrillator (ICD), this is possible in both groups, but only with backup or no pacing (e.g. in case of a nontransvenous ICD) in the AF ablation group. A total of 220 patients (110 in each group) will be enrolled (see below for sample size calculation). As it is anticipated to have significant selection bias, a patient eligibility logbook will be recorded. All patients will sign an informed consent form. Each patient's follow-up begins on the day of inclusion and concludes at study closure.

#### Endpoints

##### Primary endpoints

The study has two co-primary endpoints:

1. The composite of all-cause death or CVH (superiority hypothesis).
2. The composite of all-cause death of HFH (noninferiority hypothesis).

These endpoints will be evaluated sequentially in a hierarchical order, in order to preserve the type I error rate. The superiority hypothesis has to be fulfilled prior to evaluation of the noninferiority hypothesis (the former is felt to be most relevant and anticipated to be reached more readily and is therefore evaluated first). The main analysis of the primary endpoints is conducted in the full analysis population under application of the intention-to-treat principle, i.e. events are counted regardless of the implementation of the randomized ablation treatment.

The endpoint of CVH includes HFH or admission for any of the following: AF-related symptoms, stroke, acute myocardial infarction, cardiac implantable electronic device (CIED) interventions, redo procedures, or procedure-related complications. Admissions for crossover procedures are counted as CVH endpoints. AVNA during or following CSP implantation is not counted as an endpoint as it is an integral part of the strategy in patients randomized to the CSP + AVNA arm. HFH is defined as admission for worsening HF to a healthcare facility for > 24 h or unplanned hospital visits for HF requiring intravenous diuretics and/or adjustment of other medical HF therapy.

Predefined factors will be evaluated by subgroup analysis (e.g. age, sex, LVEF, heart rate, intrinsic QRS duration, NYHA class, duration of AF, history of AF ablation, etc.).

An independent Clinical Endpoints Committee will adjudicate the primary endpoints and will be blinded to treatment allocation whenever feasible.

##### Secondary endpoints

Individual components of the primary endpoints and a number of additional endpoints will be evaluated (duration of CVH, reintervention rate, crossovers, CIED implantation or upgrade, sinus rhythm at follow-up, LVEF (evaluated by echocardiography at the study centre), left atrial size, NYHA class, mEHRA score, and quality of life using the Minnesota Living with Heart Failure and EQ-5D-5L questionnaires). Patient-reported outcome measures (PROMs) at 1 year will be assessed by a visual analogue scale questionnaire developed for each study arm based upon PROMs of AF^43^ as well as for living with a pacemaker^44,45^ (see Supplementary material). Healthcare costs and cost-effectiveness will be analysed separately.

##### Safety endpoints

All cardiovascular adverse events related to pacemaker implantation, pacing, or ablation procedures will be recorded and adjudicated by an independant Clinical Endpoints Committee and an independent Data Safety and Monitoring Board.

### Study intervention

**Figure 1:**
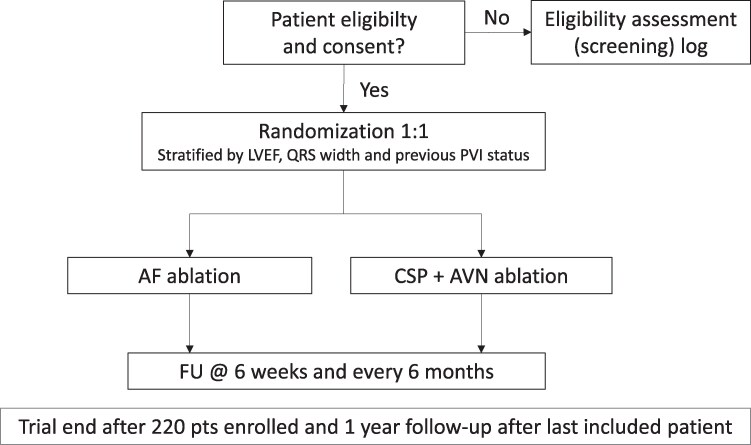
Study flowchart. Abbreviations: AF, atrial fibrillation; AVN, atrioventricular node; CSP, conduction system pacing; FU, follow-up; LVEF, left ventricular ejection fraction; PVI, pulmonary vein isolation.

Patients will be randomized 1:1 using REDcap to either AF ablation with PVI or CSP + AVNA stratified by three prognostic factors: LVEF (≤ or > 40%), QRS width (≤ or > 120 ms), and previous PVI status (first procedure or redo). The study flowchart is shown in *Figure 1*, and the clinical investigation procedures are shown in *Table 3*.

**Table 3:** Clinical investigation procedures and assessments

	Enrolment	Intervention (within 3 mo)	@ 6 wk^a^ (±2 wk)	@ 6 mo^a^ + each 6 mo (±2 wk)	@ 1 yr^a^ (±2 wk)	End of study visit
Consent form	X					
Randomization	X					
Medical history	X					
Medication	X	X	X	X	X	X
NYHA class	X	X	X	X	X	X
QOL	X				X	
mEHRA score	X	X	X	X	X	X
PROMs					X	
12-lead ECG	X	X	X	X	X	X
Echocardiogram (within 3 months before enrolment)	X				X	
Device check (CSP arm)		X	X	X	X	X
Adverse events		X	X	X	X	X
Mortality status		X	X	X	X	X
End visit form						X

^a^Counted from the date of intervention

Abbreviations: CSP, conduction system pacing; ECG, electrocardiogram; ICF, informed consent form; QOL, quality of life questionnaire (MLWHF + EQ-5D); PROMs, patient-reported outcome measures.

Ablation of AF may be performed according to the operator’s preference (e.g. using radiofrequency ablation, cryoablation, PFA, etc.) and following current European Heart Rhythm (EHRA) recommendations^46^ but should include PVI and restoration of sinus rhythm as goals. Additional lesions (e.g. lines, substrate modification, vein of Marshal ethanol infusion, etc.) may be performed according to the discretion of the operator. Rate and/or rhythm control medical therapy, anticoagulation, and heart failure treatment may be continued after the ablation procedure, as indicated. Operators must have performed >200 AF ablations as a primary operator.

Implantation of CSP (HBP or LBBAP) is currently performed in routine clinical practise^34,35,42,47^ and may be performed according to the operator’s preference and following the technique described in the EHRA consensus document on CSP implantation^47^ but should include conduction system capture (as outlined in the EHRA consensus document of CSP implantation^47^) or left ventricular septal pacing (LVSP) as a goal. With HBP, a backup ventricular pacing lead is preferred, in accordance with the current recommendations.^33,36^ A coronary sinus lead for His-optimized or left-bundle branch optimized CRT (HOT-CRT and LOT-CRT) may be implanted if deemed necessary due to suboptimal paced ECG parameters.^36^ An atrial lead may be implanted at the physician’s discretion if reversion to sinus rhythm is anticipated. If CSP implantation is unsuccessful, the physician may proceed with treatment as clinically indicated, but if possible with BiVP (which is the closest form of physiological pacing), or otherwise with RVP. The generator to be connected to the CSP lead is discretionary. Programming should be optimized to alleviate symptoms, maximize device longevity, and ensure patient safety. Operators must have performed >100 CSP implantations as primary operator.

Radiofrequency catheter AVNA may be performed as a staged procedure or during CSP implantation (also using the superior venous access) according to operator preference (and is not counted as an endpoint).

All procedures should be performed using CE-approved tools and according to instructions for use.

### Sample size calculation and statistical analysis

The event rates extracted from previous studies (see Supplementary material) were evaluated to calculate the required sample size. With a 2 to 3-year recruitment period and all patients being followed up for at least 1 year after randomization of the last patient, the expected mean follow-up duration is 2 years.

For the sample size calculation for the primary superiority hypothesis (mortality + CVH), the expected two-year event rate is 42% in the AF ablation group and 24% in the CSP + AVNA group, which corresponds with a Hazard Ratio of 0.5038. With these event rates, randomization of 2 × 105 evaluable patients provides the trial with 80% power to demonstrate the superiority of CSP and AVNA over AF ablation.

The expected 2-year event rate for the primary noninferiority hypothesis (the composite of all-cause mortality and HFH) is 18% in the AF ablation group and 12% in the CSP and AVNA groups, which corresponds to a Hazard Ratio of 0.6442. With these event rates, randomization of 2 × 105 evaluable patients provides the trial with 80% power to demonstrate the noninferiority of CSP and AVNA compared to AF ablation, with a noninferiority margin of 1.80 for the hazard ratio, which was deemed to be clinically acceptable.

To compensate for loss to follow-up, the study is designed to enrol 220 patients, with follow-up of all patients until at least one year after randomization of the last patient.

The main analysis of the primary endpoints will be conducted in all randomized subjects under application of the intention-to-treat principle at 1-year follow-up of the last enrolled patient (or earlier if recommended by the DSMB based on interim analysis of safety data). The superiority endpoint is declared if the 2-sided *P*-value from the adjusted hazard ratio between groups falls to <0.05. The noninferiority endpoint is declared if the 95%-confidence interval for the adjusted Hazard Ratio from Cox proportional hazards regression model falls below 1.8. The use of 95% confidence interval is equivalent to noninferiority testing with a one-sided type I error (*α*) of 0.025. The hypotheses of the primary endpoints will be tested in a hierarchical order in order to preserve type I error rate of 0.05, i.e. the superiority endpoint will have to be fulfilled for the noninferiority endpoint to be evaluated. There will be no formal interim analyses for early trial termination based on early claims of superiority of CSP and AVNA over PVI ablation.

All statistical analyses of secondary endpoints are descriptive using 95% confidence intervals and no formal hypothesis testing. The 95% confidence intervals are calculated without corrections for multiplicity. Time-to-event secondary endpoints will be analysed using the Kaplan–Meier curves, and hazard ratios will be calculated using Cox proportional hazards regression analysis. Hazard ratios for secondary endpoints that do not include all-cause death will be interpreted as cause-specific hazard ratios, with censoring follow-up at the time of unrelated death. The binary outcomes of the two randomized groups will be compared using logistic regression models. Quantitative secondary endpoints will be described using means and 95% confidence intervals, or medians and interquartile ranges, as appropriate. Changes in quality of life will be analysed with multivariate regression models. An exploratory Win-Ratio analysis^48^ using the components of the primary endpoints will be performed.

The study dataset may be provided to third parties for research purposes only in coded manner where no individual patient identification is possible, and upon agreement between the PIs.

## Discussion

The purpose of our study is to provide, for the first time, a head-to-head comparison of two contemporary treatment strategies for rhythm and rate/regularization control for persistent AF in HF patients. We decided to also include patients with one previous PVI, as simplification of the procedure has led to more permissive patient selection, but the clinical conundrum of whether a repeat ablation or a pace and ablate strategy should be performed remains an issue. In addition, limiting patient selection to those without a previous PVI would be restrictive and slow down enrolment.

The first patient was enrolled on 15 October 2024 and the anticipated recruitment period of the 220 patients is 2–3 years, with at least 1 year of follow-up for all patients, meaning that we expect the trial closeout to be by October 2028. Recruitment is likely to be impacted by selection bias of the site investigators as well as treatment preference by patients and referring physicians, and a patient eligibility assessment log will be maintained. The possibility to first perform a single AF ablation should however help to mitigate this issue.

We believe that the results of ABACUS will have major implications in the management of this patient population, not only in terms of impacting their clinical outcome, but also regarding healthcare costs of the two treatment strategies.

## Supplementary Material

oeag007_Supplementary_Data

## Data Availability

The data underlying this study will be shared on reasonable request to the corresponding author.

## Lead author biography

Professor Haran Burri, FESC, FEHRA, is a device specialist and electrophysiologist based at the Cardiology Department of the University Hospital of Geneva (Switzerland), where he is director of the device programme. His research has focused on device therapy and on conduction system pacing in the last years with > 250 publications. He is Past-President of the Swiss Working Group on Pacing and Electrophysiology, and is the current 2024–2026 President-Elect of the European Heart Rhythm Association (EHRA).
