# Supplementary material for: Catheter Ablation of atrial fibrillation vs. atrioventricular nodal ablation with Conduction system pacing in persistent atrial fibrillation and heart failure (ABACUS): rationale and design
Source: Eur Heart J Open. 2026 Jan 19;6(1):oeag007. doi: 10.1093/ehjopen/oeag007 (PMC12930383; doi:10.1093/ehjopen/oeag007)
Supplement: oeag007_Supplementary_Data [file oeag007_supplementary_data.zip › PROMs.pdf]

Visual Analog Scale for atrial fibrillation ablation

1. How satisfied are you with the overall result of your atrial fibrillation ablation?

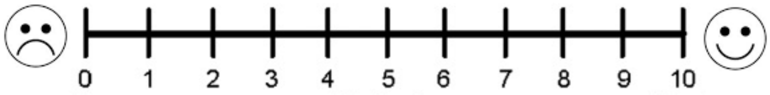

Not satisfied Very satisfied

2. To what extent has your treatment relieved your symptoms of atrial fibrillation?

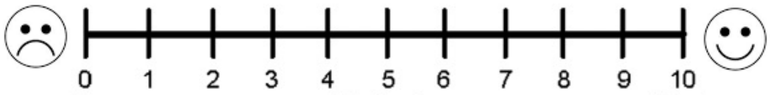

Extremely dissatisfied Extremely satisfied

3. Has your atrial fibrillation ablation impacted your hobbies/occupation?

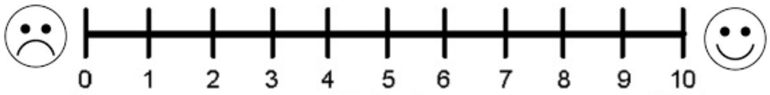

No change Improves my activities

Inversion of the scale

---

4. Are you worried about recurrence of atrial fibrillation?

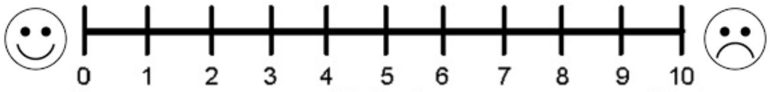

Not worried Very worried

5. Do you worry about having to take medication in case atrial fibrillation comes back??

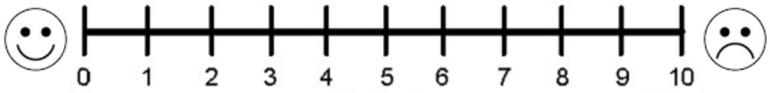

Not worried Very worried

6. Do you worry about having to perform a new intervention in case of atrial fibrillation recurrence?

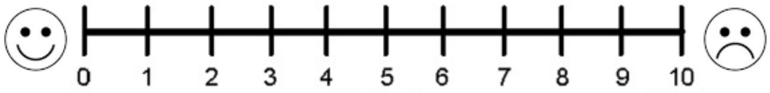

Not worried Very worried

Visual Analog Scale for Pace and Ablate

1. How satisfied are you overall with result of pacemaker implantation and nodal ablation?

0 1 2 3 4 5 6 7 8 9 10

Not satisfied — Very satisfied

2. To what extent has your treatment relieved your symptoms of atrial fibrillation?

0 1 2 3 4 5 6 7 8 9 10

Extremely dissatisfied — Extremely satisfied

3. Have the pacemaker and atrioventricular node ablation impacted on your hobbies/occupation?

0 1 2 3 4 5 6 7 8 9 10

Restricts my activities — Improves my activities

Inversion of the scale

---

4. How much pain or discomfort do you experience related to your pacemaker?

0 1 2 3 4 5 6 7 8 9 10

No pain / discomfort — Very painful/uncomfortable

5. How do you feel about the cosmetic appearance of your pacemaker?

0 1 2 3 4 5 6 7 8 9 10

Very good — Very bad

6. Are you concerned about being "dependant" on a device"?

0 1 2 3 4 5 6 7 8 9 10

Not concerned — Very concerned

7. How much concern do you feel that your pacemaker will stop working or malfunction?

0 1 2 3 4 5 6 7 8 9 10

No concern — Always concerned

8. How would you rate any sleep disturbances related to your pacemaker?

0 1 2 3 4 5 6 7 8 9 10

No sleep disturbance — Considerable sleep disturbance
