# Supplementary material for: Catheter Ablation of atrial fibrillation vs. atrioventricular nodal ablation with Conduction system pacing in persistent atrial fibrillation and heart failure (ABACUS): rationale and design
Source: Eur Heart J Open. 2026 Jan 19;6(1):oeag007. doi: 10.1093/ehjopen/oeag007 (PMC12930383; doi:10.1093/ehjopen/oeag007)
Supplement: oeag007_Supplementary_Data [file oeag007_supplementary_data.zip › Source data for sample size calculations.pdf]

ABACUS

SOURCE DATA FOR SAMPLE SIZE CALCULATIONS

Assumptions for AF ablation group are made according to data from the RAFT-AF trial,[1] which has a population anticipated to be similar to patients in ABACUS (other than older age in ABACUS). Assumptions for CSP + AVN ablation group made from Su et al.[2]

1. Summary:

Superiority endpoint of total mortality + CVH (including redo AF ablation)

	AF ablation	CSP + AVN ablation
1 year	30%	12%
2 years	42%	24%

Non-inferiority endpoint of total mortality + HFH

	AF ablation	CSP + AVN ablation
1 year	11.0%	6.0%
2 years	18.0%	12.0%

2. Event rate estimates for AF ablation group

Assumptions according to RAFT-AF[1] graphs and supplementary material.
Data were processed so that a given patient will not count towards several events (e.g. AF redo and HFH or CVH for other causes).

Assumptions:

- Higher total mortality in ABACUS due to anticipated older mean age
- Half of patients to be included in ABACUS will have had one previous AF ablation.
- Less 2-year AF redo rate in ABACUS compared to RAFT-AF due to part of the population having already had a previous AF ablation, as well as better lesion durability with new ablation technologies.

At 1 year:
- Total mortality + HFH = 10% (according to fig 3) +1% mortality accounting for older age in ABACUS =11%

- Total mortality is 3% (according to Suppl. Fig.) +1% assumption due to older population = 4%
- Incidence of CVH (<u>repeat AF ablation are not counted here</u>) is reported 12.6% at 1 year (derived from DSMB table showing 20.9% over median of 20 months), rounded up to 13%
- AF redo rate assumed at 25% (cf discussion below of table S5). Taking into account multiple events in a same patient (AF redo+HFH+other CVH), one can assume that about half of AF redos will have had other CVH events, and that only 13% of AF redos will count towards total CVH.

- Mortality + CVH (including redos) = 4+13+13=30%

At 2 years:
- Total mortality + HFH = 16% according to fig 3, +2% mortality assumption for older age=18%
- Total mortality is 6% according to Suppl. Fig. +2% mortality due to older population = 8%
- According to DSMB table 12.6%/year of CVH = rounded to 25% at 2 years (not counting redos)
- AF redo rate assumed to be 35%. One can assume that many of these patients will have multiple other events (HFH or other non-ablation CVH, e.g. for AF-related symptoms or procedural complication), and that only 9% of redos will count towards total CVH.

- Therefore, total mortality + CVH (including AF redo) at two years of 8% + 25%+ 9% = 42%

Data extraction details from RAFT-AF[1]

RAFT-AF[1]: 214 pts with AF ablation and *population similar to ABACUS except for age* (see table) *and provides the most details of endpoints in the supplementary material*.
Median follow-up 37.4 mo (Q1, Q3: 24.7, 53.7).
HF events defined as an admission to a health care facility for >24 hours or clinically significant worsening HF leading to the administration of intravenous diuretic in an emergency department or unscheduled visit to a health care provider, and an increase in chronic HF therapy. <u>Admission to the hospital for a redo AF ablation was not considered a serious adverse event.</u>
ABACUS patients are likely to be older (mean age >70 years).

Table 1. Baseline Characteristics

Characteristics	Rate control (n=197)	Ablation-based rhythm control (n=214)
Age, y, mean±SD	67.5±8.0	65.9±8.6
Female sex, n (%)	49 (24.9)	57 (26.6)
Race, n (%)		
Asian	3 (1.5)	6 (2.8)
Black	0	2 (0.9)
White	193 (98.0)	204 (95.3)
Other	1 (0.5)	2 (0.9)
Body mass index, mean±SD	30.7±6.7	30.1±6.5
Underlying heart disease, n (%)		
Ischemic	55 (27.9)	74 (34.6)
Nonischemic	142 (72.1)	140 (65.4)
New York Heart Association class, n (%)		
II	131 (66.5)	144 (67.3)
III	66 (33.5)	70 (32.7)
Time from first diagnosis of AF, mo, median (Q1, Q3)	15 (6, 48)	14.5 (7, 36)
AF type, n (%)		
High-burden paroxysmal	11 (5.6)	19 (8.9)
Persistent type 1: AF <7 days but previous cardioversion	9 (4.6)	7 (3.3)
Persistent type 2: AF ≥7 days	129 (65.5)	140 (65.4)
Long-lasting persistent AF ≥1 y	48 (24.4)	48 (22.4)
Previous cardioversion, n (%)	116 (58.9)	114 (53.3)
Cardiac implanted electric devices (all)	67 (34.0)	68 (31.8)
Implantable cardioverter defibrillator	27 (13.7)	25 (11.7)
Pacemaker	15 (7.6)	14 (6.5)
CRT-P	1 (0.5)	7 (3.3)
CRT-D	24 (12.2)	22 (10.3)
Previous coronary revascularization (coronary artery bypass graft surgery/percutaneous coronary intervention), n (%)	45 (22.8)	64 (29.9)
Hospitalization for heart failure in the previous 9 mo, n (%)	60 (30.5)	71 (33.2)

On September 25, 2017, the Data Monitoring Committee recommended that enrollment be terminated and follow-up be continued for a minimum of 2 years for all patients. This decision was based on lower-than-expected enrollment and perceived futility. Data available on all 363 patients enrolled up to that time with follow-up for a median of 19.5 months.

DSMC table

Protocol Plan vs. Results at this DMC Meeting: (Data on: **9 Sep 2017**)		
**PATIENT STATUS**	Protocol Plan	Current Performance
Randomized: Ablation (A)	300	191
Randomized: Rate Control (R)	300	172
Median Follow-Up Months (Q1,Q3)	36	A: 20.2 (10.3,38.2)<br>R: 19.6 (9.8,40.9)
**EFFICACY**	Protocol Plan	Current Performance
**Primary Composite Outcome (Unrefuted)**		
Ablation Arm	11.9%	30 (15.7%)
Rate Control Arm	17%	22 (12.8%)
Hazard Ratio (95% CI)	0.70	1.32 (0.76,2.28)
Other pre-specified outcomes, sub-groups or secondary analyses? ❍No ●Yes		
Are Statistical Warning Rules due for application at this time? ❍No ●Yes		
**OTHER EFFICACY OUTCOMES**		
**Secondary Outcome (Unrefuted)**	Ablation Arm	Rate Control Arm
All-cause Mortality	17 (8.9%)	12 (7.0%)
CV Mortality	11 (5.8%)	8 (4.7%)
All-cause Hospitalization	61 (31.9%)	37 (21.5%)
HF Hospitalization	20 (10.5%)	16 (9.3%)
CV Hospitalization	40 (20.9%)	28 (16.3%)
**Subgroups (Unrefuted)**	Ablation Arm	Rate Control Arm

CV hospitalization of 20.9% at median 20 months follow-up = 12.6%/year

Table S5. RAFT-AF Ablation Procedural details

	**Ablation group (n=205)**
Time of ablation from randomization (BL) – days (N=205) Mean ± SD	44.7±54.0
Successful pulmonary vein isolation	100%
Patients with one ablation – no. of pts	128 (62.4%)
One repeat procedure – no. of pts	69 (33.7%)
Two repeat procedures – no. of pts	8 (3.9%)
Three repeat procedures- no of pts	0
Time between initial procedure and 1st repeat ablation –Mean ± SD (days)	417.5±336.1

Thus 62.4+3.9=37.6% of patients had at least one redo over a mean follow-up of 417 days (1.14 years), yielding a re-ablation rate of approximately 33% at 1 year. However, assuming that half of patients to be included in ABACUS will have had one previous AF ablation (with a reduced risk for requirement for subsequent redos), and that new

technologies may reduce these recurrence rates, one can assume that redos will be about 25% at one year and 35% at 2 years.

Follow-up median 37.4 months

Table S7. RAFT-AF Serious Adverse Event Details

N (%)	Rate control	Ablation-based rhythm control	P value*
Classification	N=197	N=214	
All events	99 (50.3%)	102 (47.7%)	0.5997
Cardiovascular	68 (34.5%)	66 (30.8%)	0.4270
Angina	2 (1.0%)	3 (1.4%)	1.0000
Atrial fibrillation	2 (1.0%)	18 (8.4%)	0.0005
Atrial flutter	1 (0.5%)	8 (3.7%)	0.0384
Heart failure decompensation	48 (24.4%)	38 (17.8%)	0.0999
Myocardial infarction-non-fatal	0	4 (1.9%)	0.1245
Ventricular tachycardia	9 (4.6%)	4 (1.9%)	0.1183
Ventricular fibrillation	2 (1.0%)	0	0.2291
Stroke	5 (2.5%)	5 (2.3%)	1.0000
Transient ischemic attack	2 (1.0%)	0	0.2291
Other cv	13 (6.6%)	9 (4.2%)	0.2815

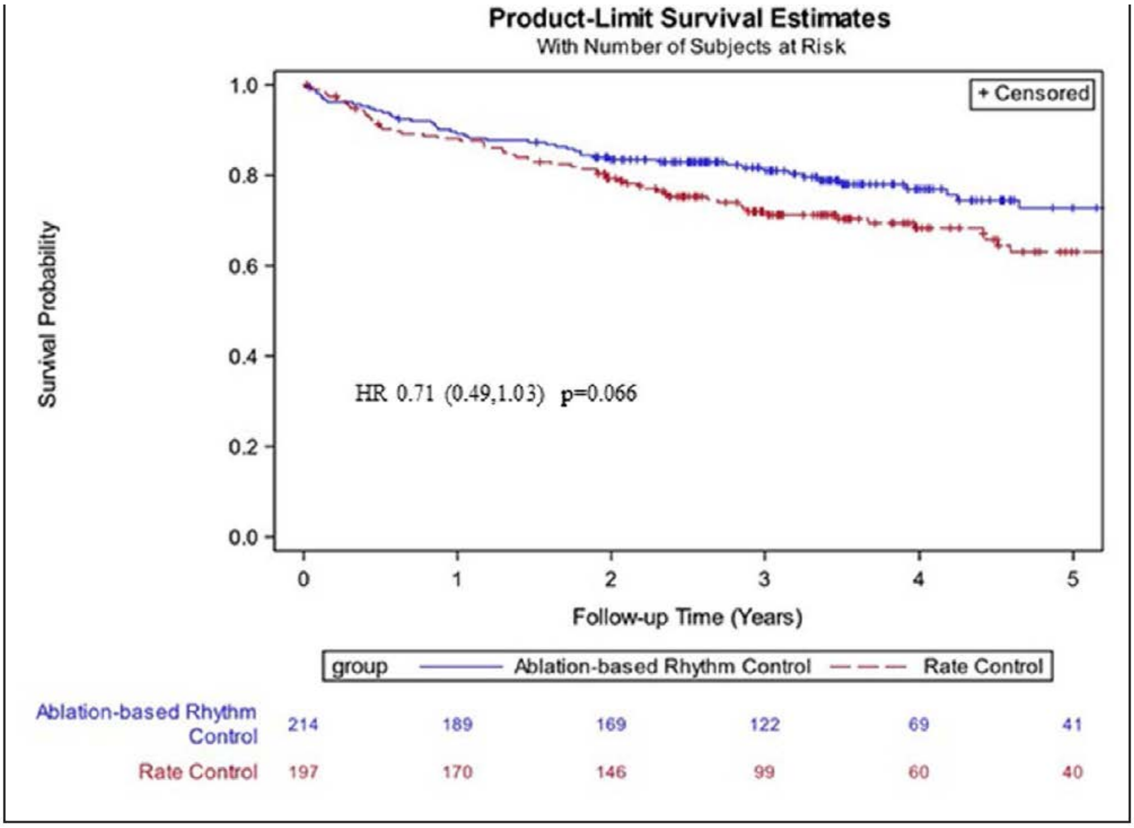

Figure 3. Freedom from all-cause mortality or heart failure event.

Ablation arm : 10% @ 1 year; 16%@ 2 years; 19% @ 3 years

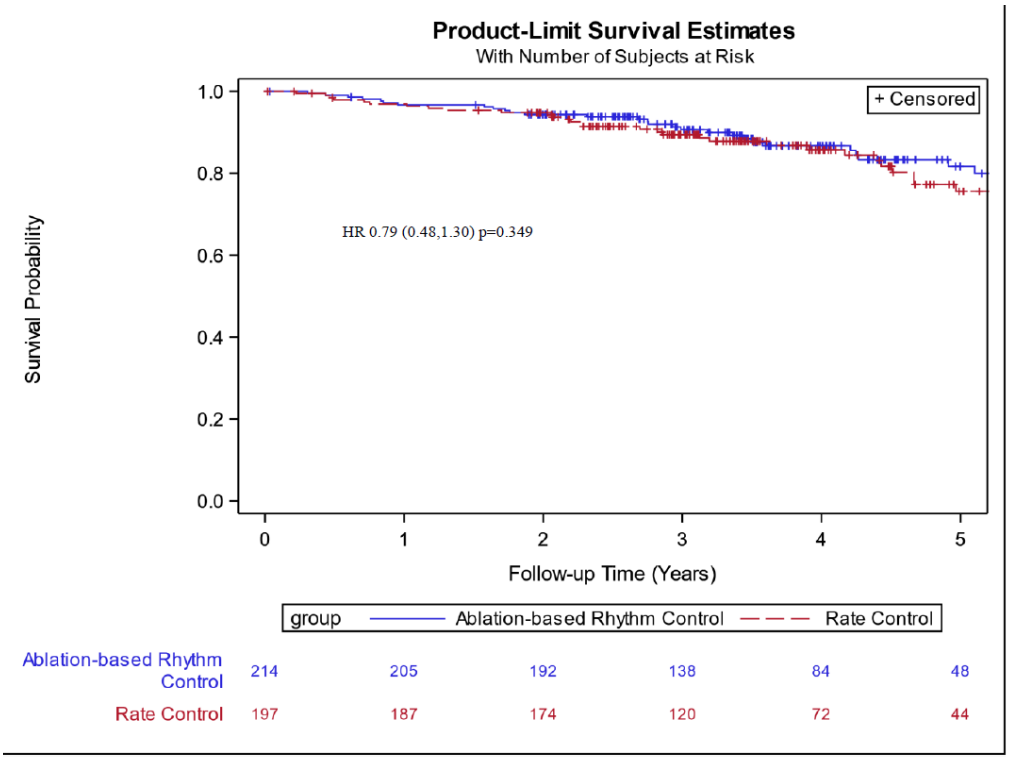

Ablation arm : Mortality 3% @ 1 year; 6% @ 2 years; 9%@ 3 years

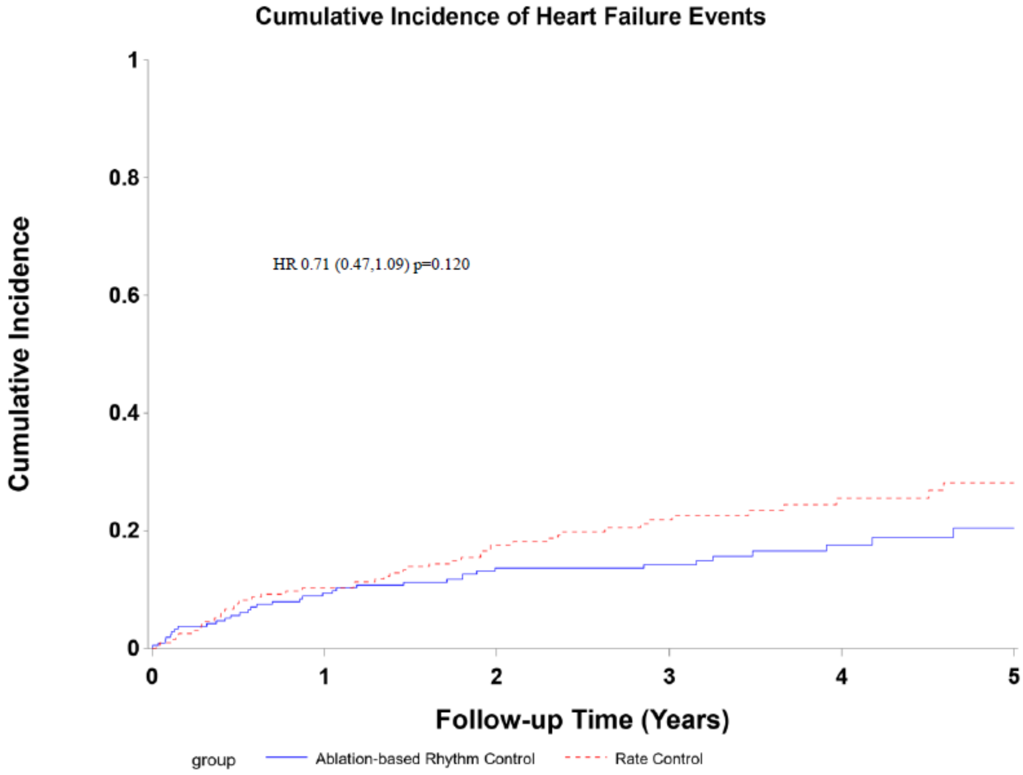

Ablation arm: HF @1 yr = 10 %; @ 2 yrs = 13%; @ 3 yrs = 14%

Other AF ablation studies (not considered for ABACUS sample size calculations):

CASTLE-AF[3]: 179 pts with AF ablation, highly selected population, with either paroxysmal (30%) or persistent (70%) AF, with LVEF $\leq$0.35 and equipped with ICD. *Therefore likely to be sicker than in ABACUS.*

CASTLE HTx[4]: 194 patents with end-stage heart failure referred for heart transplantation with paroxysmal (30%) or persistent (70%) AF and equipped with a CIED. Mean age ~63 years and mean LVEF ~27%. *Therefore sicker than in ABACUS.*

CABANA HF subgroup analysis[5]: 378 pts with AF ablation and NYHA II-IV heart failure; paroxysmal AF in 29%; median LVEF 55%. *Therefore likely to be less sick than in ABACUS.*

3. Event rate estimates for CSP + AVN ablation group

Data are scant and with small population size, making it difficult to make accurate projections for ABACUS. Data were extracted from Su et al.[2]

Assumptions

- According to figure from Su et al.[2] Mortality + HFH = 6%, at 1 year and 12% at 2 years.
- No studies reported CVH. Data of non-HF, non-device-related cardiovascular events (angina + VT/VF+stroke/TIA+other CV=33/197=16.8%) from rate control group SAE table S7from RAFT-AF[1] were used and averaged for the median 3 year (37.4 months) follow-up, rounded down to 5%/year (to account for multiple events in a same patient).
- Most available data of device complications from CSP+AVN ablation is for HBP. However, it is anticipated that the great majority of patients in ABACUS will be implanted with LBBAP, with lower revision rates than for HBP. Device revisions are projected at 1% /year.

Therefore:
- mortality + CVH at 1 year = 6+5+1=12%
- mortality + CVH at 2 years = 12+10+2=24%

Data extraction details from Su et al.[6].

Population of 81 HF pts with narrow QRS and HBP (mean age 70 yrs, LVEF 0.45, 89% NYHA II/III, 11% NYHA IV).

During median FU 3 yrs, 14 pts died and 7 pts had HFH. There were 3 lead revisions and 1 pocket infection.
According to figure, mortality and HFH was 6% at 1 year, 12% at 2 years, and 26% at 3 yrs:

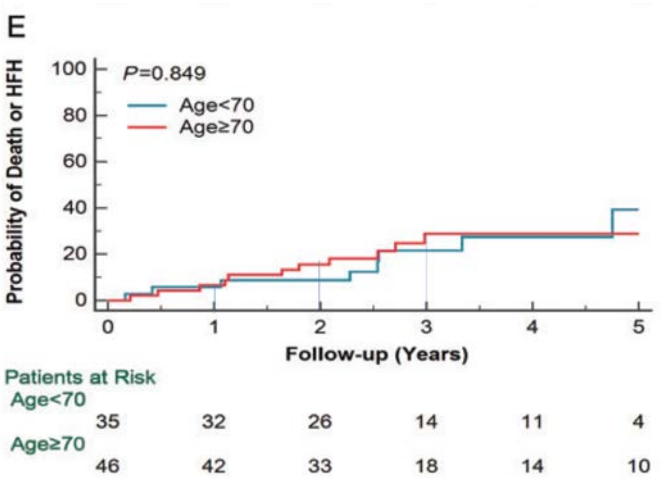

4. Other studies on CSP + AVN ablation

Wu et al.[7] : 170 pts with LVEF<0.50 (mean age 69 years, mean LVEF 0.34, HBP 62%, LBBP 38%) with at least 1 year follow-up.
During a median follow-up of 18.9 (11.9, 30.5) months, a total of 22 subjects (12.9%) experienced 14 deaths (8.2%) and eight heart failure hospitalizations (4.7%).
According to figure, mortality and HFH or transplantation of narrow QRS group was 4% at 1 year and 11% at 2 years (similar to the 12% seen in Su et al[6]). and 21% at 3 years

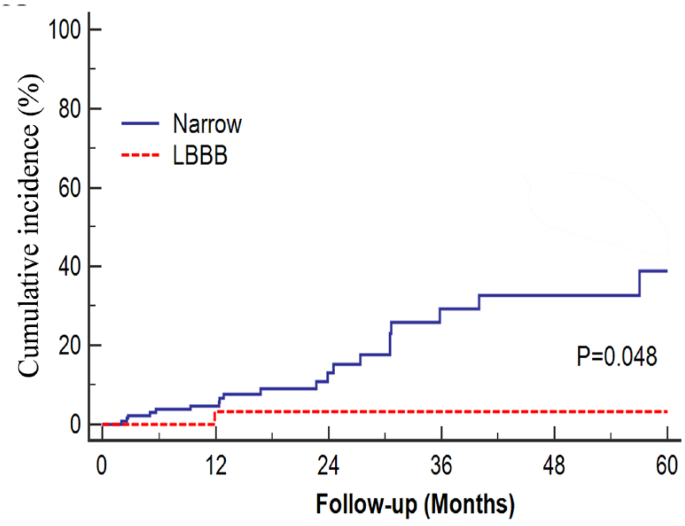

Huang et al.[8] 42 pts with HrEF or HpEF and HBP (mean age 72 yrs, mean LVEF 0.45). Median 20 month FU, during which there were 2 (4.8%) deaths and 2(4.8%) device-related hospitalizations. At 1 year, there were 2 (4.8%) HFH.

ALTERNATIVE AF[9]. Randomized crossover of HBP with BiVP for 9 months each in pts with LVEF <0.40 (mean age 64 yrs, LVEF 0.33). Only 38/50 pts finally included. 3 HFH (1

during HBP and 2 during BiVP).
The only study to report QOL (SF-36).

Table 2 NYHA functional class, BNP, and QoL at baseline, and after HBP and BVP

				HBP vs BVP		HBP vs baseline		BVP vs baseline	
	Baseline (n = 38)	HBP (n = 38)	BVP (n = 38)	Difference (95% CI)	*P* value	Difference (95% CI)	*P* value	Difference (95% CI)	*P* value
NYHA functional class	2.82 ± 0.65	1.50 ± 0.60	1.58 ± 0.64	−0.1 (−1.0 to 0.2)	.317	−1.3 (−1.6 to −1.0)	<.001	−1.2 (−1.5 to −0.9)	<.001
lgBNP concentration	2.9 ± 0.6	2.6 ± 0.6	2.6 ± 0.5	0 (−0.1 to 0.1)	.727	−0.3(−0.4 to −0.2)	<.001	−0.3(−0.5 to −0.2)	<.001
QoL score (SF-36) (n = 27)									
SF-36 PF	76.3 ± 17.3	83.2 ± 15.3	82.0 ± 15.1	1.1 (−7.2 to 9.5)	.786	−6.9 (−64.81.1)	.088	−5.7 (−11.7 to 0.2)	.058
SF-36 PR	29.8 ± 42.1	86.1 ± 20.0	83.3 ± 17.0	2.8 (−6.5 to 12.0)	.542	−56.3 (−75.7 to −36.9)	<.001	−53.5 (−71.2 to −35.8)	<.001
SF-36 BP	82.6 ± 23.7	83.3 ± 19.1	85.1 ± 24.5	−1.8 (−8.2 to 4.7)	.579	−0.7 (−13.2 to 11.8)	.906	−2.5 (−16.7 to 11.7)	.722
SF-36 GH	59.4 ± 15.4	65.9 ± 15.5	61.3 ± 17.3	4.6 (−4.2 to 13.5)	.291	−6.5 (−13.5 to 0.5)	.067	−1.9 (−11.7 to 8.0)	.701
SF-36 VT	64.8 ± 12.4	65.6 ± 16.9	65.6 ± 16.3	0 (−8.3 to 8.3)	.996	−0.8 (−8.3 to 6.8)	.838	−0.7 (−9.3 to 7.8)	.860
SF-36 SF	65.4 ± 23.5	75.3 ± 20.8	75.7 ± 23.5	−0.4 (−10.1 to 9.2)	.931	−9.9 (−21.71.9)	.097	−10.3 (−24.13.5)	.137
SF-36 RE	43.2 ± 45.1	91.4 ± 17.5	91.3 ± 19.8	0 (−11.0 to 11.0)	.999	−48.2 (−67.3 to −29.0)	<.001	−48.2 (−69.6 to −26.7)	<.001
SF-36 MH	67.6 ± 17.1	73.9 ± 15.1	73.0 ± 22.2	0.9 (−8.1 to 9.9)	.841	−6.4 (−13.6 to 0.9)	.083	−5.5 (−16.4 to 5.4)	.312

Values are given as mean ± SD unless otherwise indicated.

CI = confidence interval; QoL = quality of life; SF-36 = 36-Item Short Form Health Survey (PF = Physical functioning; PR = Role-physical; BP = Bodily pain; GH = General health; VT = Vitality; SF = Social functioning; RE = Role-emotional; MH = Mental health); other abbreviations as in Table 1.

References

[1] Parkash R, Wells GA, Rouleau J, Talajic M, Essebag V, Skanes A, et al. Randomized Ablation-Based Rhythm-Control Versus Rate-Control Trial in Patients With Heart Failure and Atrial Fibrillation: Results from the RAFT-AF trial. *Circulation* 2022; **145**: 1693-1704.

[2] Su L, Cai M, Wu S, Wang S, Xu T, Vijayaraman P, et al. Long-term performance and risk factors analysis after permanent His-bundle pacing and atrioventricular node ablation in patients with atrial fibrillation and heart failure. *Europace* 2020; **22**: ii19-ii26.

[3] Marrouche NF, Brachmann J, Andresen D, Siebels J, Boersma L, Jordaens L, et al. Catheter Ablation for Atrial Fibrillation with Heart Failure. *N Engl J Med* 2018; **378**: 417-427.

[4] Sohns C, Fox H, Marrouche NF, Crijns H, Costard-Jaeckle A, Bergau L, et al. Catheter Ablation in End-Stage Heart Failure with Atrial Fibrillation. *N Engl J Med* 2023.

[5] Packer DL, Piccini JP, Monahan KH, Al-Khalidi HR, Silverstein AP, Noseworthy PA, et al. Ablation Versus Drug Therapy for Atrial Fibrillation in Heart Failure: Results From the CABANA Trial. *Circulation* 2021; **143**: 1377-1390.

[6] Su L, Wang S, Wu S, Xu L, Huang Z, Chen X, et al. Long-Term Safety and Feasibility of Left Bundle Branch Pacing in a Large Single-Center Study. *Circ Arrhythm Electrophysiol* 2021; **14**: e009261.

[7] Wu S, Su L, Vijayaraman P, Zheng R, Cai M, Xu L, et al. Left Bundle Branch Pacing for Cardiac Resynchronization Therapy: Nonrandomized On-Treatment Comparison With His Bundle Pacing and Biventricular Pacing. *Can J Cardiol* 2021; **37**: 319-328.

[8] Huang W, Su L, Wu S, Xu L, Xiao F, Zhou X, et al. Benefits of Permanent His Bundle Pacing Combined With Atrioventricular Node Ablation in Atrial Fibrillation Patients With Heart Failure With Both Preserved and Reduced Left Ventricular Ejection Fraction. *J Am Heart Assoc* 2017; **6**.

[9] Huang W, Wang S, Su L, Fu G, Su Y, Chen K, et al. His Bundle Pacing vs Biventricular Pacing Following Atrioventricular Node Ablation in Patients with Atrial Fibrillation and Reduced Ejection Fraction: A Multicenter, Randomized, Crossover Study. The ALTERNATIVE-AF trial. *Heart Rhythm* 2022.
